# Dry season habitat use of fishes in an Australian tropical river

**DOI:** 10.1038/s41598-019-41287-x

**Published:** 2019-04-05

**Authors:** K. Keller, Q. Allsop, J. Brim Box, D. Buckle, D. A. Crook, M. M. Douglas, S. Jackson, M. J. Kennard, O. J. Luiz, B. J. Pusey, S. A. Townsend, A. J. King

**Affiliations:** 1 Research Institute for the Environment and Livelihoods, Engineering Health Science & Environment, Charles Darwin University, Darwin, NT 0909 Australia; 2 Department of Primary Industry and Resources, Berrimah Road, Berrimah, NT 0828 Australia; 3 Department of Environment and Natural Resources, Alice Springs, NT 0870 Australia; 4 School of Earth and Environment, University of Western Australia, Perth, WA 6009 Australia; 5 Australian Rivers Institute, Griffith University, Kessels Road, Nathan, QLD 4111 Australia; 6 Water Resources Division, Department of Environment and Natural Resources, Palmerston, NT 0830 Australia

## Abstract

The modification of river flow regimes poses a significant threat to the world’s freshwater ecosystems. Northern Australia’s freshwater resources, particularly dry season river flows, are being increasingly modified to support human development, potentially threatening aquatic ecosystems and biodiversity, including fish. More information is urgently needed on the ecology of fishes in this region, including their habitat requirements, to support water policy and management to ensure future sustainable development. This study used electrofishing and habitat survey methods to quantify the dry season habitat use of 20 common freshwater fish taxa in the Daly River in Australia’s wet-dry tropics. Of twenty measured habitat variables, water depth and velocity were the two most important factors discriminating fish habitat use for the majority of taxa. Four distinct fish habitat guilds were identified, largely classified according to depth, velocity and structural complexity. Ontogenetic shifts in habitat use were also observed in three species. This study highlights the need to maintain dry season river flows that support a diversity of riverine mesohabitats for freshwater fishes. In particular, shallow fast-flowing areas provided critical nursery and refuge habitats for some species, but are vulnerable to water level reductions due to water extraction. By highlighting the importance of a diversity of habitats for fishes, this study assists water managers in future decision making on the ecological risks of water extractions from tropical rivers, and especially the need to maintain dry season low flows to protect the habitats of native fish.

## Introduction

Freshwater ecosystems are threatened around the world by flow modification^1^. Flow modification can be attributed to a number of human-related activities including urbanisation, industrialisation, mining and agriculture arising from anthropogenic structures such as dams, reservoirs, levee’s and channelization^1–3^. Flow is a major driver of physical habitat in rivers and streams; affecting the biotic composition, distribution and diversity of all aquatic life^4^. Alterations to river flow can therefore affect the availability of habitat quality and quantity, and in turn can negatively impact the assemblage composition and abundance of aquatic species^4,5^.

Stream fishes are closely associated with physical habitat attributes at a range of spatial scales^6,7^. Variations in water depth and velocity have been hypothesised by various studies as the key factors influencing habitat use of fishes, with physical attributes such as substrate composition, submerged vegetation, wood and root masses also being important^8–10^. The availability and types of physical habitat attributes can vary within and between river systems, and can influence species composition, distribution and abundances, due to differences in ecological requirements for food, spawning sites and/or refuge^11–13^. Previous studies have also classified groups of species into habitat use guilds to summarise the relationships among taxa, and to explore the influence of habitat on assemblage dynamics, not just individual species (e.g. Grossman & Freeman^14^; Leonard & Orth^15^; Aadland^16^; Lamouroux & Souchon^17^). These studies often incorporate water depth, velocity and physical habitat features (e.g. substrate composition) in assigning taxa to certain habitat guilds. For some species, habitat use can also change ontogenetically (e.g. Leonard & Orth^15^; Aadland^16^); thus life stage may be considered as an important factor in assessment of species-habitat associations.

Hydrological and water quality variability can influence the availability and type of aquatic habitats present for fish to use. In tropical regions, seasonality and interannual variability of the flow regime may be the most important temporal influence on fish distribution and abundance^18–20^. In the wet-dry tropics of Northern Australia, this effect of flow is particularly evident at the end of the dry season when water levels are at their lowest due to reduced rainfall, and factors such as competition and predation can strongly influence fish community structure^21,22^. In these systems, aquatic organisms have developed different modes of adaptation (e.g. life history, behaviour, morphology) in response to the seasonal timing and predictability of flow events^4,23^. Several studies indicate that the combination of persistent, predictable flows and habitat complexity are important in promoting and maintaining species specialisation and diversification in tropical rivers^10,24,25^.

Northern Australian rivers are recognised both nationally and internationally for their high ecological and cultural value^26,27^, and support higher aquatic species diversity than temperate Australian rivers^28–30^. However, recent efforts to develop northern Australia (e.g. expanded agricultural and mining interests) may result in increased demand for water resources, potentially reducing the diversity and abundance of aquatic habitats and associated biota, including fish^18,31,32^. Deleterious impacts on subsistence fisheries are of particular concern to the numerous Indigenous traditional owner groups of the region^33^. Knowledge of fish habitat requirements are common considerations in management and assist in amelioration actions such as environmental flow rules^34–36^. However, our knowledge of many aspects of the ecology of northern Australian fishes, including habitat requirements, is currently sparse; and therefore our ability to inform water policy and management about fish requirements is limited.

This study aims to explore the dry season habitat use of freshwater fishes in the Daly River, Northern Territory; a perennial river with a largely unmodified flow regime in Australia’s wet-dry tropics. The study uses a data-rich, multi-year dataset to quantify fish-habitat associations during the dry season. It was hypothesised that (i) fish taxa would be strongly associated with flow-related characteristics such as water depth and current velocity, (ii) there are distinct fish habitat guilds associated with specific mesohabitat features (e.g. riffles, pools) and (iii) ontogenetic habitat changes would occur for some species. The implications of our findings are discussed with regards to future water management in tropical rivers.

## Methods

### Study area and focal reach

**Figure 1:**
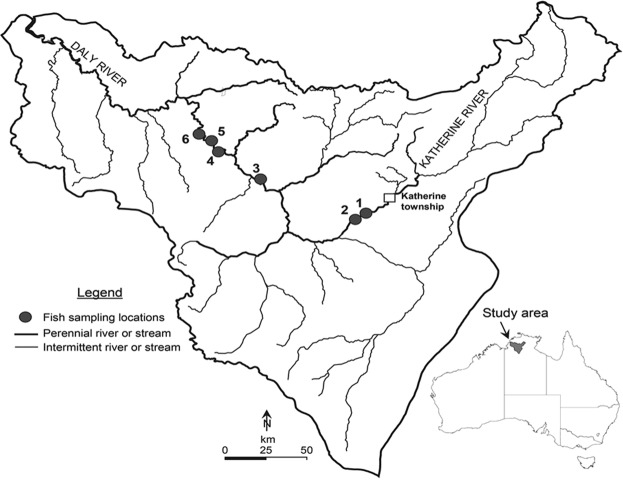
Location of fish sampling locations in the Daly River catchment. Fish sampling sites are indicated with site identification numbers (1–6). Refer to Table 1 for site details. The inset shows the location of the study area in northern Australia.

The Daly River catchment (mean annual streamflow 8653 GL^37^) is located in the wet-dry tropical climate region of northern Australia (Fig. 1), covers ~53,000 km^2^, and has a largely intact savannah ecosystem throughout the catchment^38^. The catchment’s population is 10,000 people, of whom 28% are indigenous^39^. The Katherine (largest tributary) and the Daly Rivers are considered to be in relatively good ecological condition^38,40^. All rivers in the catchment are unregulated (no dams or weirs), but some groundwater extraction occurs for agriculture and water supply and this is likely to increase in the future^18^. The rivers have a characteristic wet-dry tropical flow regime, with predictable and large wet season flows with high interannual variability^41^. Mean annual rainfall averages 1070 mm^42^ across the catchment and is highly seasonal, with the majority falling during the wet season months (November-April), and negligible rainfall during the dry season (May–October). Flow in the Daly River and some tributaries is perennial, with significant groundwater inputs coming from two underlying aquifers, resulting in a lengthy period of continuous and stable dry season base flows (May-November). The Daly and Katherine rivers are sand-bed rivers that contain dispersed bedrock outcrops and gravel bars; river banks are typically steep, rising 15–20 m above the river bed^42,43^. Riparian vegetation in the catchment is largely intact and natural, consisting of *Eucalyptus* woodlands, *Melaleuca* forests and closed monsoon rainforests^44^.

This study occurred along a 120 km long reach of river from near Katherine township downstream to Oolloo crossing (Fig. 1). Six sites that were accessible by boat were selected; these sites represent the available habitat types for fish within the reach (Table 1).

**Table 1:** Details of six sampling sites in the Daly River catchment.

Site identification number	Site name	Dates sampled
1	Katherine River at Galloping Jacks	Aug 2006-Oct 2011
2	Katherine River 1 km downstream of Galloping Jacks	Aug 2006-Oct 2015
3	Daly River at Claravale	July 2006-Oct 2015
4	Daly River upstream of Oolloo Crossing #1	July 2006-June 2012
5	Daly River upstream of Oolloo crossing #2	Sept 2013-Oct 2015
6	Daly River at Oolloo Crossing	July 2006-Oct 2015

### Fish and habitat sampling

Fish were surveyed biannually in both the early and late dry season, over a 10-year period from 2006–2015 (*n* = 89 sampling events in total). A detailed description of fish sampling methods can be found in Stewart-Koster *et al*.^19^ and Chan *et al*.^18^, but is briefly summarised here. Within each sampling site (500–1000 m reach length, 5–100 m wetted width), fish were sampled at discrete multiple locations (shots or replicates) using a boat or backpack electrofishing (pulsed DC), depending on water depth. Electrofisher settings were adjusted to maximise efficiency of collecting fish with minimum power. At least 15 electrofishing shots of five minute elapsed duration were undertaken for each sampling event, and shots were stratified to ensure each available habitat type within each site was sampled at least once. Prior examination of sampling efficiency has revealed that 15 shots yields an accurate estimate of species composition and assemblage structure within each site (Kennard *et al*. unpubl. data). Electrofishing shots were conducted in as homogenous area as possible and averaged 77 m (±48 SD) in length (range = 5–263 m). At the completion of each electrofishing shot, fish captured were counted and identified to species, measured (standard length (SL) in mm) and returned alive to the point of capture. The total number of fish from each species observed and reliably identified but not caught during each electrofishing shot was also recorded. Catch data were converted to catch per unit effort (CPUE—total number of individuals caught and observed per electrofishing shot). Some species captured during sampling were separated into juvenile or adult age classes (Table 2), where the minimum length used for juvenile determination for these species was <150 mm SL, except *Lates calcarifer*, where the minimum length was <300 mm SL.

**Table 2:** Fish taxa recorded during the 2006–2015 study period including age classes used in habitat analyses. Two taxa (*Ambassis* sp. and *Neosilurus hyrtlii*) were excluded since there was little explanatory power for these taxa (see Suppl. Table 1 in Supplementary Material).

Family	Taxon	Common name	Age classes	Mean CPUE ± SE (# individuals/shot)	Relative abundance (% of total)	Mean standard length (mm) ± SD
Ariidae	*Neoarius* spp.	Forktailed catfish	NA	0.8 ± 0.1	3.16	275 ± 78
Atherinidae	*Craterocephalus stercusmuscarum*	Flyspecked hardyhead	NA	0.3 ± 0.07	1.23	37 ± 9
Atherinidae	*Craterocephalus stramineus*	Blackmast	NA	5.4 ± 0.34	21.36	33 ± 8
Apogoniidae	*Glossamia aprion*	Mouth almighty	NA	0.8 ± −0.05	3.15	53 ± 24
Belonidae	*Strongylura kreffti*	Longtom	NA	0.1 ± 0.01	0.56	259 ± 118
Clupeidae	*Nematalosa erebi*	Bony bream	NA	2 ± 0.19	7.79	175 ± 61
Eleotridae	*Mogurnda mogurnda*	Northern trout gudgeon	NA	0.1 ± 0.02	0.32	43 ± 10
Eleotridae	*Oxyeleotris lineolata*	Sleepy cod	Juvenile Adult	0.3 ± 0.02 0.2 ± 0.02	1.02 0.83	147 ± 68
Eleotridae	*Oxyeleotris selheimi*	Giant gudgeon	Juvenile Adult	0.1 ± 0.01 0.1 ± 0.01	0.47 0.22	122 ± 74
Gobiidae	*Glossogobius* sp.	Goby	NA	0.1 ± 0.01	0.52	99 ± 25
Latidae	*Lates calcarifer*	Barramundi	Juvenile Adult	0.60 ± 0.04 0.4 ± 0.03	2.44 1.48	291 ± 103
Mugilidae	*Planiliza* (=*Liza*) *ordensis*	Diamond mullet	NA	1.2 ± 0.09	4.59	258 ± 69
Melanotaeniidae	*Melanotaenia australis*	Rainbowfish	NA	4.9 ± 0.27	19.53	39 ± 9
Plotosidae	*Neosilurus ater*	Black catfish	NA	0.6 ± 0.05	2.56	275 ± 78
Soleidae	*Leptachirus triramus*	Freshwater sole	NA	0.6 ± 0.07	2.29	59 ± 9
Terapontidae	*Amniataba percoides*	Barred grunter	NA	2.3 ± 0.13	9.1	63 ± 18
Terapontidae	*Hephaestus fuliginosus*	Sooty grunter	Juvenile Adult	1.2 ± 0.08 0.2 ± 0.02	4.75 0.66	83 ± 52
Terapontidae	*Leiopotherapon unicolor*	Spangled perch	NA	1.7 ± 0.11	6.63	68 ± 20
Terapontidae	*Syncomistes butleri*	Butler’s grunter	Juvenile Adult	0.3 ± 0.03 0.4 ± 0.04	1.17 1.59	145 ± 71
Toxotidae	*Toxotes chatareus*	Sevenspot archerfish	NA	0.6 ± 0.05	2.58	86 ± 42

The unit for species abundance is CPUE (total number of individuals per electrofishing shot). The mean CPUE ± standard error (SE) and relative abundance of all taxa was calculated as the total number of individuals per shot across all six sites.

A total of 20 variables describing fish habitat characteristics was measured within the area of each discrete fishing location (sampling shot) by a second field team. A full description of the methods employed in describing habitat structure is available in Kennard *et al*.^45^. Briefly, five replicate measures within each electrofishing shot were measured for depth (initially using a staff, then a Garmin 150 Fish Finder) and mean water velocity (Swoffer™ 2000, and Hach™ FH950 flow meter, measured as 20% total depth). The total percentage contribution of each mesohabitat type (i.e. riffles, runs, pools) and substrate composition (modified Wentworth scheme- see Pusey *et al*.^46^) was visually estimated within each shot (summed to 100%). The proportional contributions of a range of submerged structures (including wood, macrophytes, root masses etc. Table 3) were also visually estimated for each shot (contributions of these variables were not required to add up to 100%). The fish collected within each electrofishing shot were assigned the habitat characteristics of that shot and these data were used to examine the local habitat use of each taxon.

**Table 3:** Habitat variables estimated or measured in each electrofishing shot location over the 2006–2015 study period used in analyses (adapted from Pusey *et al*. 2004).

Variable	Description
Depth (cm)	Vertical distance from water surface to channel bottom. Measured depth ranged from 0–300 cm
Mean water velocity (cm/sec)	Water velocity measured at 0.6 x water depth. Measured mean velocity ranged from 0–128 cm/s
**Substrate composition (% surface area)**	**Visually estimated**
Mud	<0.06 mm (particle size)
Sand Fine gravel	0.06–2 mm 2–16 mm
Coarse gravel	16–128 mm
Rock	>128 mm
Bedrock	
**Submerged structures (% surface area)**	**Visually estimated**
Submerged wood	Submerged wood (>1 cm minimum stem diameter)
Leaf litter	Accumulations of leaf litter and fine woody material (<1 cm stem diameter)
Aquatic vegetation	Aquatic macrophytes, emergent vegetation (e.g. sedges, rushes) and submerged marginal vegetation (e.g.terrestrial grasses)
Submerged overhanging vegetation	Overhanging terrestrial vegetation in contact with water surface (e.g. tree branches/leaves).
Filamentous algae	
Undercut bank (% bank)	Percentage of river bank overhanging water by at least 30 cm, and no more than 10 cm above water surface
Root mass (% bank)	Percentage of river bank with submerged bankside root masses

### Data analysis

The data were filtered to remove 26 rare taxa (i.e. those taxa present in <5% of samples and/or with <50 individuals collected in total). *Neoarius graeffei*, *N*. *bernei* and *N*. *midgleyi* were combined to genus level (i.e. *Neoarius* spp.), due to either low counts or unreliable identification to species. This resulted in a total of 22 taxa suitable for analysis with six taxa separated into juvenile and adult age classes (Table 2). For the entire study period, 54,739 taxa-habitat data points were available for analysis.

Fish habitat variables were assessed for their potential to influence the abundance of each fish taxon across electrofishing shots using boosted regression trees (BRTs). Modelling can improve ecological understanding and allow evaluation of generalizability^47^, and thus has advantages over traditional approaches to quantifying habitat use (e.g. by comparing habitat availability to frequency of use to infer habitat selection or preference)^48,49^. Tree-based modelling approaches such as boosted regression trees (BRTs) are widely used for developing predictive species-environment relationships^50^ and have been applied to quantify fine-scale fish habitat use (e.g. Sharma *et al*.^51^, Greenwood^52^). BRTs are a decision tree based method which uses a boosting technique to combine large numbers of relatively simple tree models adaptively to optimize predictive performance^53^. Some advantages of BRTs include their ability to model complex and nonlinear relationships and to accommodate missing data and large numbers of potential predictor variables by ignoring non-informative variables^53^. BRTs also perform better than other regression techniques under conditions of high multicollinearity, especially when a large number of variables are analysed.

We assumed a Poisson distribution for the response variable in all models since our data is composed of individual fish counts in each shot. The best combination of parameters required by BRTs (learning rate, tree complexity and bag fraction) was identified for every model using cross-validation^52^. Cross-validation was automatically repeated for learning rates from 0.001 to 0.05 (steps of 0.002), tree complexities of 1–3 and bag fractions of 0.5 and 0.75, which span the range of likely optimal values^52^. The combinations that generated the lowest mean cross-validation deviances, calculated from at least 1000 trees, were used for the final models. Following the derivation of full models with all variables, models were investigated to establish whether irrelevant predictors could be removed (procedure as detailed in Elith *et al*.^52^).

The percentage relative contributions of each habitat variable, including the effect of season (early or late dry season) were calculated from the BRTs for each taxon. The relative contribution of a variable describes the proportion of variation in the data explained by that variable relative to all other variables in the model, scaled to 100^53^. For each taxon, partial-dependence functions were fitted for the top two most influential variables to visualise relationships between fish abundances and each variable. The fitted function for a given variable incorporates the average effect of all other variables (Elith *et al*. 2008), with positive fitted function values suggesting an increase in abundance, while negative values suggest a decrease. Following initial BRT analyses, upon examination of percent deviance explained for each model, two taxa (*Ambassis* spp. and *Neosilurus hyrtlii*) with low calculated percentage deviance (<20%) were removed from further consideration (Table 4). Hence BRTs for a total of 20 taxa with five taxa separated into adult and juvenile categories are reported, noting that each model has different percent deviance and hence explanatory power (Table 2).

**Table 4:** Number of trees and the percentage deviance explained as calculated for fish taxa from the Boosted Regression Tree models.

Species	Number of trees	Percentage deviance explained (%)
*C*. *stercusmuscarum*	3500	61.0
*H*. *fuliginosus J*	5350	49.5
*P*. *ordensis*	7850	49.1
*L*. *triramus*	5000	44.7
*L*. *unicolor*	6050	44.6
*S*. *butleri A*	5200	43.7
*O*. *lineolata A*	5350	40.8
*N*. *erebi*	5650	39.6
*N*. *ater*	4900	39.3
*A*. *percoides*	7950	38.7
*L*. *calcarifer J*	4450	38.4
*G*. *aprion*	7950	37.2
*Glossogobius* sp.	2700	36.5
*Neoarius* spp.	3250	36.3
*T*. *chatareus*	5750	35.7
*O*. *selheimi J*	3800	35.4
*L*. *calcarifer A*	3450	35.2
*S*. *butleri J*	4750	34.3
*C*. *stramineus*	4850	34.1
*O*. *lineolata J*	4500	33.4
*O*. *selheimi A*	2100	31.5
*M*. *mogurnda*	1600	31.0
*H*. *fuliginosus A*	2050	25.4
*M*. *australis*	4050	24.7
*S*. *kreffti*	2100	20.2
***Ambassis*** **sp**.	**1250**	**3**.**8**
***Neosilurus hyrtlii A***	**1000**	**3**.**5**
***Neosilurus hyrtlii J***	**650**	**0**.**88**

The higher the percentage deviance explained, higher is the predictive value of the model. Taxa highlighted in bold were excluded due to a very low calculated percentage deviance (<20%). J = Juveniles, A = Adult.

Hierarchical cluster analysis with complete linkage distance was used to group fish taxa abundance and habitat variables into habitat guilds. The number of classes (guilds) in the clustering dendrogram was determined visually in a way that allowed for a biological interpretation. Pairwise comparisons were conducted between each taxon and all habitat variables using Pearson’s correlation coefficient. All analyses were performed in R version 3.3.2^54^ using the ‘gbm’ package version 2.1.3^55^ and the ‘dismo’ package version 1.1–4^56^ for the BRT models plus customised code written and described by Elith *et al*.^52^, and the ‘hclust’ function of ‘vegan’ package version 2.4-4^57^ for the cluster analysis.

### Ethical Statement

All methods were carried out in accordance with ethical guidelines and regulations and approved by Charles Darwin University’s Committees for Animal Ethics and Human Research Ethics. Fish sampling was also conducted under NT Fisheries S17 Fisheries Act Permit.

## Results

**Figure 2:**
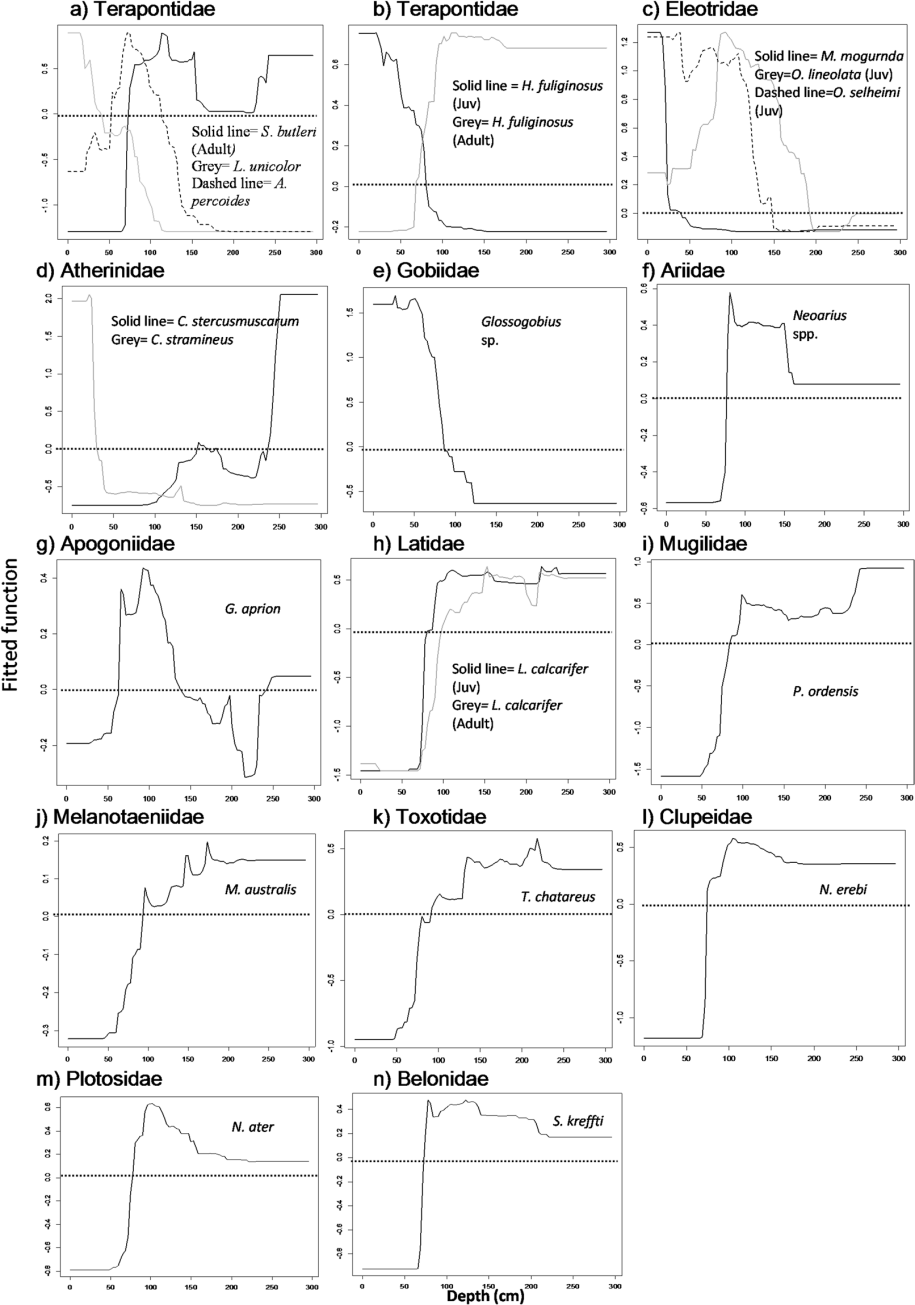
(**a**–**n**) Partial dependence plots showing relationships between taxa abundance and depth as the first or second most important variable in the BRT models, plots grouped into families. Note different y axis ranges. Refer to Table 4 for the percentage relative contribution of each variable for all taxa. Taxa were grouped into family categories for comparison between species and ontogeny (juvenile/adult). Dotted line highlights zero on the fitted function axis. Positive fitted function values above the dotted line shows high abundance and low values below this line show the opposite. Juvenile and adult *H*. *fuliginosus* (**b**) are separated from other Terapontids for clarity.

Twelve habitat variables were identified as being the top two most important contributors to the BRT models for the 20 taxa (Table 5). Seasonal variation in habitat use was minimal, with this factor having a low relative influence in BRT models for all taxa (Table 5). Water depth was identified as the top contributor for 16 taxa (Fig. 2, Table 5). Taxa that were most abundant in shallow habitats (0–100 cm) included *L*. *unicolor*, juvenile *H*. *fuliginosus*, *M*. *mogurnda*, juvenile *O*. *selheimi*, *C*. *stramineus*, and *Glossogobius* sp. In contrast, both *C*. *stercusmuscarum* and *P*. *ordensis* were more likely to occur in deeper habitats (>200 cm). The remaining taxa, *A*. *percoides*, adult *S*. *butleri*, adult *H*. *fuliginosus*, juvenile *O*. *lineolata*, *Neoarius* spp., *G*. *aprion*, juvenile and adult *L*. *calcarifer*, *M*. *australis*, *T*. *chatareus*, *N*. *erebi*, *N*. *ater*, and *S*. *kreffti*, were most abundant in moderate to deep habitats (100–200 cm).

**Table 5:** Percentage relative contribution of each variable for all taxa as calculated from the Boosted Regression Tree models.

Taxa	Depth	Velocity	Mud	Sand	Fine gravel	Coarse Gravel	Rock	Bedrock	Submerged wood	Leaf litter	Aquatic Veg.	Submerged overhang Veg.	Fil.algae	Und.cut banks	Root mass	Season	Guild type
*Neoarius* spp.	*16*.*7*	**24**.**3**	0.8	8.6	0	0.6	2.5	1.9	6.8	15.9	2.3	11	3.3	3.3	2.8	0	I
*G*. *aprion*	**17**.**6**	*12*.*6*	8.4	5.8	2.6	2.7	1.9	2.7	7.2	6.4	9	0	2.4	5.9	11.1	3.8	I
*M*. *australis*	**20**.**9**	*19*.*4*	6.6	3.8	0	2.2	0	2.8	5.9	5.3	3.8	3	3.2	2.9	15.1	2.9	I
*N*. *erebi*	**18**.**4**	10.8	5.7	0	3.3	3.5	6.4	7.1	*14*.*8*	8.5	4.2	2.6	3	4.1	2.6	0	I
*N*. *ater*	**25**.**6**	*13*.*2*	1.9	5.4	3.3	2.4	0	2.6	8.2	7.8	8.1	4.4	3	7.8	2.4	4.1	I
*O*. *lineolata* J	*15*.*8*	12.2	1.6	3.6	2	1.7	2.1	5.1	9.7	7.6	2.5	2.8	2.2	5	**25**.**7**	2.3	I
*O*. *lineolata* A	12.6	*15*.*3*	2.5	5.7	0.8	0	0	6	4.5	9.9	4.6	1.8	2.2	9.1	**20**.**1**	3.8	I
*S*. *butleri* A	**24**.**7**	*14*.*6*	8.4	9.1	1.6	3.4	1.2	3.3	3	6.7	4.6	6.1	0	2.8	8.5	0	I
*T*. *chatareus*	**24**.**3**	*11*	7.1	5.5	1	2.5	0	1.5	10.3	4.9	5.6	10.8	1.7	6.4	6.1	1.4	I
*H*. *fuliginosus* A	**19**.**3**	13.7	1.6	4.7	4.2	*17*.*7*	0.9	0	10.1	1.3	4.5	0	12.5	1.3	4.4	2.8	II
*L*. *calcarifer* J	**26**	15.9	2.5	2.4	1	0	6.1	7.8	*19*.*1*	3.3	4.9	1.3	0	2.5	1.5	5.7	II
*L*. *calcarifer* A	**26**.**2**	13.7	5.8	5.2	4.6	7.7	0	0.9	*23*.*7*	1.1	1.8	2	2.6	3.6	0.9	0	II
*P*. *ordensis*	*18*.*8*	**25**.**7**	0.9	9.5	1.3	4.8	7.1	2.3	7.7	8.7	1.8	2	0	1.9	3.7	2.4	II
*O*. *selheimi* A	7.4	7.8	2.8	0.9	1.2	0.2	0	0	**39**.**8**	5.1	3.7	*23*.*5*	0.9	4.6	1.7	0.2	II
*S*. *kreffti*	**28**.**1**	8	2.9	4.1	2.5	0.9	0.7	0.6	4.2	3	*13*.*5*	11.6	12	1.6	6.6	0.5	II
*A*. *percoides*	**22**.**8**	15.6	1.8	2.4	2.8	1.5	1.6	17.1	6.5	18.6	4.2	1.7	1.4	1.1	2.1	0	III
*C*. *stercusmuscarum*	**44**.**9**	3.4	0.1	0.6	0.4	2.2	1.2	*20*.*8*	1.1	4.9	2.7	0	3.2	10	1.5	3.3	III
*C*. *stramineus*	**30**.**4**	*17*.*7*	0	1	1.7	6.3	0.5	2.7	1.4	0.7	15	0	8.8	2	1.7	10.7	III
*L*. *triramus*	15.1	5	0.1	**32**.**4**	*22*.*5*	3.9	0	4.3	6.2	2.2	2.5	0.6	0.3	1.1	0.7	2.8	III
*M*. *mogurnda*	**42**.**1**	*21*.*2*	0.2	1.4	6.7	16	2.2	0.2	2.8	5.2	1.2	0	0.2	0	0.1	0.2	III
*O*. *selheimi* J	*11*.*9*	10.6	7.8	3.5	1.4	0	1.9	11.7	11.2	2.8	0	7.5	1.9	7.1	**16**.**9**	2.8	III
*Glossogobius* sp.	**28**.**9**	13	0	1.5	4.1	*20*.*1*	3.8	10.8	1.9	0.1	2.6	0	1	0.3	0.7	11.2	IV
*H*. *fuliginosus* J	*16*.*2*	**32**.**4**	0	7.4	1.1	13.3	11.4	3.1	7.7	2.4	1.1	0	1.5	0.8	0.7	1	IV
*L*. *unicolor*	**38**.**9**	8.2	1.6	3	1.3	4.3	2.6	8.9	3.7	*9*.*2*	5.2	0	4.2	0	1.6	6.5	IV
*S*. *butleri* J	8.1	*23*.*8*	0	4.4	1.6	10.4	**28**.**1**	4.6	4.6	1.8	2.1	0.6	7.5	0	0.6	0.9	IV

The top contributing variables are highlighted in bold and the second most contributing variables are underlined. J = Juvenile, A = Adult, Av = average, Veg. = vegetation, Fil. = filamentous, Und.cut banks = undercut banks. Fish habitat guild types: (i) fishes occupying deep pools containing root masses and undercut banks, (ii) large-bodied fishes occupying deep pools containing wood, (iii) a mixed habitat use guild of small-bodied fishes, and (iv) small-bodied fishes occupying shallow riffles with high water velocities and coarse substrates.

**Figure 3:**
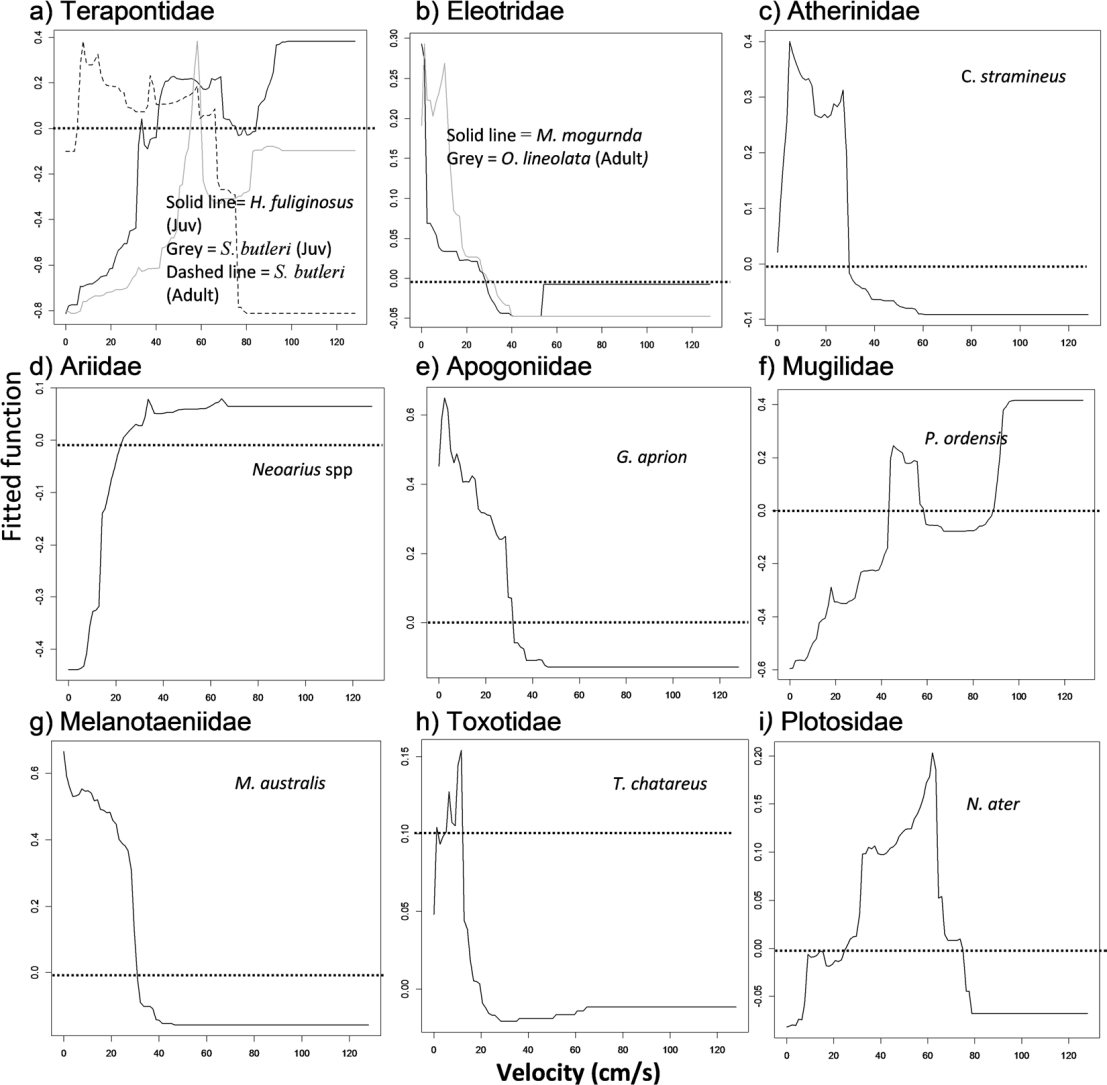
(**a**–**i**) Partial dependence plots showing relationships between taxa abundance and velocity as the first or second most important variable in the BRT models, plots grouped into families. Note different y axis ranges. Refer to Table 4 for the percentage relative contribution of each variable for all taxa. Taxa were grouped into family categories for comparison between species and ontogeny (juvenile/adult). Dotted line highlights zero on the fitted function axis. Positive fitted function values above the dotted line shows high abundance and low values below this line show the opposite.

Velocity was the second most important contributor to the BRT models, occurring as one of the top two most important contributor for 12 taxa overall, and was the top habitat contributor for three taxa (Fig. 3, Table 5). Taxa that were most abundant in low velocity habitats (0–30 cm/s) included adult *O*. *lineolata C*. *stramineus*, *G*. *aprion*, *M*. *australis*, *M*. *mogurnda*, *T*. *chatareus* and adult *S*. *butleri*. Both juvenile *H*. *fuliginosus* and *P*. *ordensis* were most abundant in fast flowing habitats (>90 cm/s). The remaining taxa, juvenile *S*. *butleri*, *N*. *ater* and *Neoarius* spp., were most abundant in habitats with moderate to high velocities (30–90 cm/s).

**Figure 4:**
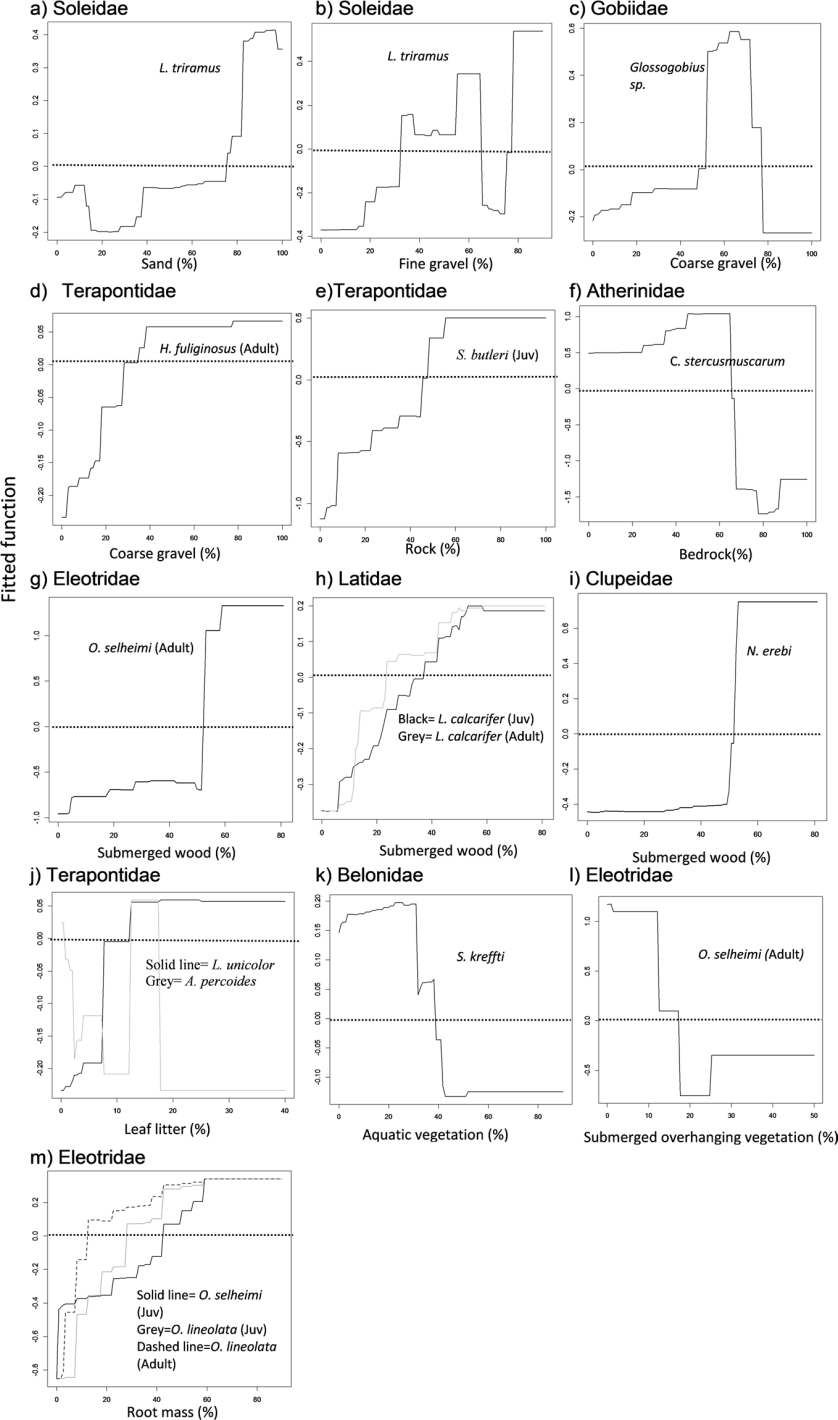
(**a**–**m**) Partial dependence plots showing relationships between taxa abundance and other variables which were identified as the first or second most important variable in the BRT models, plots grouped into families. Note different y axis ranges. Refer to Table 4 for the percentage relative contribution of each variable for all taxa. Taxa were grouped into family categories for comparison between species and ontogeny (juvenile/adult). Dotted line highlights zero on the fitted function axis. Positive fitted function values above the dotted line shows high abundance and low values below this line show the opposite.

Submerged wood, root mass, leaf litter, rock, sand, fine gravel, coarse gravel, bedrock, filamentous algae and overhanging vegetation were the next most important contributors to the BRT models (Fig. 4, Table 5). The abundance of both juvenile and adult *L*. *calcarifer*, adult *O*. *selheimi*, and *N*. *erebi*, were all higher in habitats with over 50% submerged wood. Similarly, the abundance of both juvenile and adult *O*. *lineolata*, as well as juvenile *O*. *selheimi* was higher in habitats with over 40% root mass. Likewise, the abundance of *L*. *unicolor* and juvenile *S*. *butleri* was also higher in habitats with over 15% leaf litter and 40% rock, respectively. The abundance of *L*. *triramus* was higher in habitats with over 80% sand and fine gravel, respectively. Similarly, the abundance of *Glossogobius* sp. and adult *H*. *fuliginosus* was higher in habitats with 50–75% and over 40% coarse gravel, respectively. In contrast, the abundance of *A*. *percoides*, *C*. *stercusmuscarum*, *S*. *kreffti* and adult *O*. *selheimi* was lower in habitats with over 15% leaf litter, 60% bedrock, 30% aquatic vegetation and 10% overhanging vegetation, respectively.

**Figure 5:**
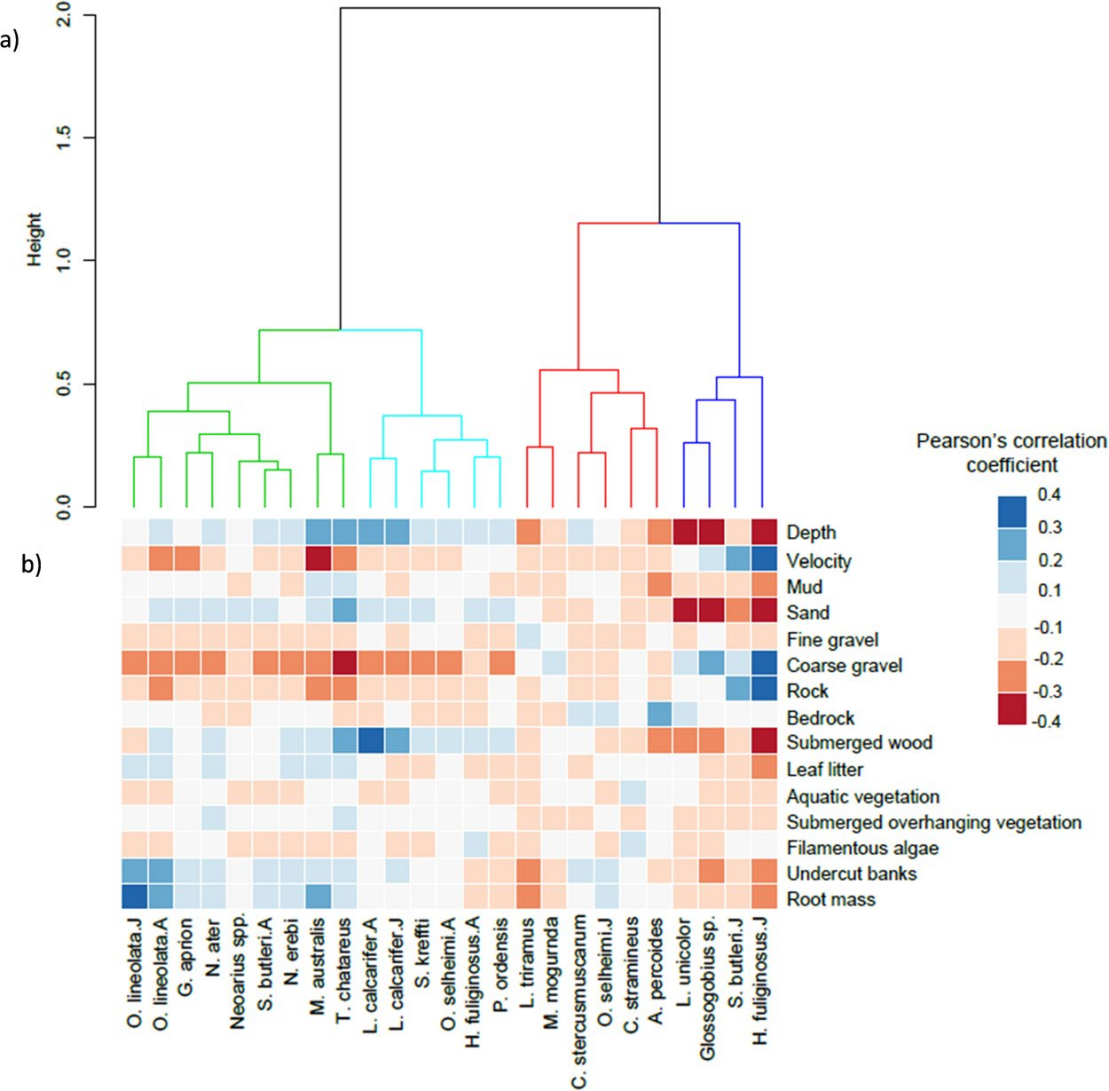
Hierarchical cluster analysis with pairwise comparisons between taxa and habitat variables as used in the BRT models, displaying (**a**) four distinct habitat-use guilds and (**b**) the strength of the positive (blue) and negative (red) associations with each habitat variable, using the Pearson’s correlation coefficient. Habitat Guilds (**a**): Green = fishes occupying deep pools containing root masses and undercut banks (Guild I), Light blue = large-bodied fishes occupying deep pools containing wood (Guild II), Red = a mixed habitat use guild of small-bodied fishes (Guild III), Dark blue = small-bodied fishes occupying shallow riffles with high water velocities and coarse substrates (Guild IV); A = Adult, J = Juvenile.

Cluster analysis of the habitat data revealed four distinct habitat-use guilds (Fig. 5a). We interpreted these groups qualitatively, using information on taxon body size (Table 2) and the taxon correlations with each habitat variable (Fig. 5b). The four habitat use guilds comprised: Guild I: fishes occupying deep pools containing root masses and undercut banks, Guild II: large-bodied fishes occupying deep pools containing wood, Guild III: a mixed habitat use guild of small-bodied fishes, and Guild IV: small-bodied fishes occupying shallow riffles with high water velocities and coarse substrates.

Taxa associated with Guild I had a range of body sizes and included both juveniles and adults of O. *lineolata*, *M*. *australis*, *G*. *aprion*, adult *S*. *butleri*, *N*. *erebi*, *N*. *ater*, *Neoarius* spp. and *T*. *chatareus* (Fig. 5b). Taxa within Guild II were large-bodied fishes including both juvenile and adult *L*. *calcarifer*, adult *H*. *fuliginosus*, adult *O*. *selheimi*, *S*. *kreffti* and *P*. *ordensis*. Guild III consisted of six small-bodied taxa with a range of habitat associations. This included *L*. *triramus* which was associated with shallow habitats and higher densities of fine gravel and sand, *M*. *mogurnda* which was associated with higher densities of coarse gravel, both *C*. *stercusmuscarum* and juvenile *O*. *selheimi* which were associated with higher bedrock cover, undercut banks and root masses; *C*. *stramineus* was associated with increasing densities of aquatic vegetation and filamentous algae, and *A*. *percoides* was associated with shallow habitats with increasing bedrock cover. Guild IV consisted of small-bodied fishes occupying shallow riffles with high water velocities and coarse gravel and/or rock substrates (Fig. 5b). This included juvenile *H*. *fuliginosus*, juvenile *S*. *butleri*, *Glossogobius* sp. and *L*. *unicolor*.

## Discussion

This study used a multi-year and data-rich dataset to quantify the dry season habitat use of freshwater fishes in an Australian wet-dry tropical river. Although many taxa displayed distinct associations with the measured habitat variables, depth and velocity were the key habitat variables for most of the taxa examined (16 and 12 taxa, respectively). Other important habitat variables included submerged wood, root masses, vegetation and sediment type. The analysis also identified four distinct fish habitat guilds according to body size and habitat associations. Whilst it is important to also consider taxon-specific differences in habitat use, the use of fish habitat guilds here may have advantages for habitat management in taxa rich systems such as tropical rivers, and also for forecasting habitat use in other systems or similar taxa (e.g. Leonard & Orth^15^).

Depth and velocity are often identified as important factors in determining fish assemblage structure in rivers (e.g. this study; Jackson *et al*.^8^; Kennard *et al*.^58^). These factors characterise the geomorphological complexity of rivers^59^ and hence the availability of habitats for fish; they are also likely to influence fish behavioural characteristics, such as swimming type, reproductive and feeding behaviours. Not surprisingly then, the depth and velocity requirements of fish are common considerations in river habitat and flow management actions, such as environmental flow determinations (e.g. Bunn & Arthington^4^; Poff *et al*.^60^) and habitat restoration activities^61^. In this study, 15 taxa were associated with moderately deep and low velocity habitats or pools. Pool habitats may have higher species diversity relative to other habitat types, because: (i) they are likely to be more effectively buffered against changes in habitat structure, water level and water quality^58,62^, (ii) they contain higher structural diversity (submerged wood, undercut banks, root masses, etc.) for feeding and shelter^63^, and (iii) they usually cover a greater surface area compared to other mesohabitats, such as riffles and runs^9^.

Four taxa had higher abundance in riffle habitats, including *L*. *unicolor*, *Glossogobius* spp. and juveniles only of *S*. *butleri* and *H. fuliginosus* (Guild IV). Juveniles of the terapontids *H*. *fuliginosus* and *S*. *butleri* demonstrated ontogenetic habitat shifts, with juveniles using riffle habitats before moving to deeper, slow-flowing pool habitats as adults (deep, structured pools habitat guild, see below). These findings confirm previous research and observations for these two species^18,19,29,64^. Riffle habitats may be providing a suitable refuge habitat for these four small-bodied taxa from piscivorous and avian predators, and/or discrete feeding or reduced competition with larger individuals (e.g. Rosenfeld & Boss^65^). Both *Glossogobius* spp. and *L*. *unicolor* were grouped into the riffle-dwelling habitat guild, due to their association with shallow depths and coarse gravel. This supports in part previous research by Rayner *et al*.^24^ that suggested that *Glossogobius* sp. 1 (identified in their study) was a fluvial specialist, preferring high velocity, shallow water habitats. Our analysis also provides further evidence that *L*. *unicolor* prefers shallow habitats and coarse gravel substrates, but with little velocity preference, thus this species may utilise broader habitat types with various velocities compared to the three riffle-dwelling species grouped in this guild.

In this study, the juveniles and adults of *L*. *calcarifer*, adult *O*. *selheimi* and adult *H*. *fuliginosus*, *S*. *kreffti* and *P*. *ordensis*, showed a positive association with deep, densely structured habitats composed of submerged wood (Guild II). A number of previous studies have shown that riverine fish assemblages are influenced by the physical structural complexity of river channels, including the presence of large submerged woody debris and instream vegetation^66^. Pettit *et al*.^63^ examined the distribution and movement of large woody debris and its importance as a fish habitat in the Daly River. Using a sub-set of the same fish data analysed in the current study, they demonstrated that fish species richness, diversity and abundance were not correlated with the proportion of wood present at a reach scale. However, juveniles and adults of *L*. *calcarifer* and adults of *H*. *fuliginosus* were strongly associated with wood cover^63^. Large submerged wood is considered to be an important contributor to habitat heterogeneity, through providing overhead cover which reduces predation risk, and also camouflage for predators to ambush their prey, among other functions^66^.

Fishes associated with pools that contain root masses and undercut banks (Guild I) included a range of taxa and life stages, including *O*. *lineolata*, *G*. *aprion*, *N*. *ater*, *N*. *erebi*, *M*. *australis*, *T*. *chatareus* and adult *S*. *butleri*. These taxa were all positively associated with a high density of both root masses and undercut banks, except *Neoarius* spp., which was more strongly associated with sandy substrates and moderate depths. The presence of both undercut banks and root masses serve as important cover for *O*. *lineolata*^29^, which was found to have a strong association with this habitat type in this study. Rainbowfish (*Melanotaenia* sp.), have previously been recorded as abundant in habitats composed of both root masses and undercut banks, as well as leaf litter^46^. Mouth almighty, *G*. *aprion*, a small-bodied predatory fish, may be associated with this habitat type in order to hide and ambush its prey^22,67^. Mouth almighty have previously been classified as a microhabitat generalist^24^, but has also been described as having a close association with root masses, leaf litter and aquatic vegetation^22,67^. Undercut banks have also been suggested as important daytime resting habitat for taxa within the Plotosidae and Terapontidae families^68^, which supports the findings for *N*. *ater* and adult *S*. *butleri* in this study. In contrast, the presence of overhanging vegetation may be more important for *T*. *chatareus*, since *s*urface-dwelling and terrestrial invertebrate prey which occur in this vegetation type feature heavily in the diet of this species^67^. The presence of root masses, undercut banks and vegetation are suggested to function as an important refuge and/or foraging habitat for some species^46,68^. Indeed, many of the taxa in this habitat guild are regular prey of *L*. *calcarifer*^69^, which supports the hypothesis that the taxa in this guild are using these habitats as a predator refuge. Studies from other systems have also shown that species of small-bodied fishes (e.g. cyprinids) rarely co-exist in the same habitat type as larger predatory species, such as northern pike, *Esox lucius*^70^, and smallmouth bass, *Micropterus dolomieu*^71^. This further supports the predator refuge hypothesis.

The fourth habitat guild (Guild III) grouped six small-bodied taxa into a mixed habitat guild. Both *M*. *mogurnda* and *C*. *stercusmuscarum*, which had low associations with all habitat features in this study, have previously been shown to occur across varying substrate types^67^, while *C*. *stercusmuscarum* has been described as a habitat generalist^24^. Likewise *Oxyeleotris* sp. is described as a habitat generalist with a preference for abundant cover^29,46^, and juveniles of *O*. *selheimi* in this study were found to have a slight positive association with both root masses and undercut banks. *Amniataba percoides* was found to have a strong association with bedrock substrate in this study, but has been previously described as associated with a variety of substrates^67^. In comparison, *L*. *triramus* in this study was positively associated with both sandy and fine gravel substrates, this supports previous observations of the species being associated with sandy slackwater habitats^29^. *Craterocephalus stramineus* has previously been described as a riffle-dwelling species^22^, however we found that this species was associated with aquatic vegetation and filamentous algae.

The habitat use differences observed in this study may be due to differences among rivers and over time, or potentially this study’s coarser assessment of habitat use across a 5 minute shot length; i.e. fish may be captured in a rarer, smaller habitat type within the whole habitat sampling shot, and therefore specific associations may be overlooked. Indeed, this is an important limitation of our study and we recommend that future studies should focus on more detailed microhabitat assessments if finer resolution is important, as well as to confirm habitat use or preference. Furthermore, it is important to note that this study only examined day-time patterns of habitat use. Thus a range of complementary methods which may be used to determine spatial and temporal habitat use include snorkelling, underwater video surveys, and/or biotelemetry (e.g. Winter^72^; Lucas & Baras^73^).

Ontogenetic habitat shifts by fishes often occur in rivers (e.g. Scott & Nielsen^74^; Schiemer *et al*.^75^; King^76^), presumably because the size of an individual influences its ability to respond to velocity, seek shelter from predation and use other resources^77^. In this study, three species (*H*. *fuliginosus*, *S*. *butleri*, and *O*. *selhemi*) demonstrated distinct habitat changes from juvenile to adult life stages. As previously discussed, juvenile *H*. *fuliginosus* and *S*. *butleri* utilised riffle habitats and then shifted to deeper, slow flowing pools as adults. Similar ontogenetic shifts in habitat use have been observed in other species including brown trout, *Salmo trutta*, where young-of-the-year trout inhabited fast-flowing riffles, while larger, older trout occupied deeper and slower flowing habitats^78^. In this study, juvenile *O*. *selheimi* was associated with habitats dominated by aquatic vegetation, undercut banks and root masses (mixed habitat guild, see above), before switching to habitats with increased levels of submerged wood as adults (deep, structured pools habitat guild, see above). Conversely, our findings showed no change in habitat use between juvenile and adult life stages for *L*. *calcarifer* or *O*. *lineolata*. Whilst the number of species demonstrating ontogenetic habitat changes could be viewed as low (only three out of five species where life stages were categorised), we did not sample larval or early juvenile life stages in this study, and further research on the habitat use of these important life stages in wet-dry tropical rivers is needed.

The patterns of habitat use revealed in this study improve our understanding of the habitat requirements of a range of fish taxa during the dry season in northern Australian rivers. The study quantified fish-habitat use associations for a large number of common taxa, and identified four distinct habitat use guilds. The fish-habitat guild approach is useful in summarising and simplifying communication of fish habitat use requirements to managers and the broader community. After further exploration of the proposed fish-habitat guilds, the approach may also be useful for predicting habitat requirements in similar systems with little ecological knowledge.

While a range of habitat variables were important habitat descriptors for individual taxa and size classes, unsurprisingly water depth and velocity were key factors influencing dry season habitat use of fish in this system. Many studies have emphasised the maintenance of natural seasonal flow patterns for the persistence of freshwater fish assemblages and the diversity of riverine mesohabitats^4,58,79,80^. In perennial wet-dry river systems, the prolonged low water period during the dry season (~May-October), maintains critical riverine longitudinal connectivity and a range of shallow and deeper habitats that sustain aquatic ecosystems (this study^31,63,81^). Shallow habitats such as riffles are likely to be particularly vulnerable to natural or artificial reductions in water levels^18,31^, and hence changes in flow or dewatering of these habitats during the dry season could reduce connectivity, constrain fish movement and have particularly strong impacts on specific riffle dwellers such as *L*. *unicolor*, *Glossogobius* spp., and juvenile *H*. *fuliginosus* and *S*. *butleri* (this study^18^). These results, coupled with data on the extent of riffle habitat area at various water levels, would enable predictions to be made about the extent of riffle habitat at different water levels, and hence predict the impact on riffle-dwelling species. Incorporation of this type of information into water planning is critical for future decision making regarding the management of environmental water in these systems.

## Data Availability

Data is available through Charles Darwin University’s Research webportal (https://researchers.cdu.edu.au/).
